# Combining multiple family-based association studies

**DOI:** 10.1186/1753-6561-1-s1-s162

**Published:** 2007-12-18

**Authors:** Hua Tang, Jie Peng, Pei Wang, Marc Coram, Li Hsu

**Affiliations:** 1 Department of Genetics, Stanford University School of Medicine, 300 Pasteur Drive, Stanford, California 94305, USA; 2 Department of Statistics, University of California, Davis, California 95616, USA; 3 Division of Public Health Sciences, Fred Hutchinson Cancer Research Center, 1100 Fairview Avenue North, Seattle, Washington 98109, USA

## Abstract

While high-throughput genotyping technologies are becoming readily available, the merit of using these technologies to perform genome-wide association studies has not been established. One major concern is that for studies of complex diseases and traits, the whole-genome approach requires such large sample sizes that both recruitment and genotyping pose considerable challenge. Here we propose a novel statistical method that boosts the effective sample size by combining data obtained from several studies. Specifically, we consider a situation in which various studies have genotyped non-overlapping subjects at largely non-overlapping sets of markers. Our approach, which exploits the local linkage disequilibrium structure without assuming an explicit population model, opens up the possibility of improving statistical power by incorporating existing data into future association studies.

## Background

The past few years have seen a rapid development in high-throughput genotyping platforms, making genome-wide association studies technologically feasible. Yet the merit of using these technologies to perform genome-wide association studies has not been established [1]. Insufficient sample size is a particular concern in genome-wide association studies for several reasons. First, the genotyping cost, even though decreasing, nonetheless puts a pressure to reduce the number of individuals assayed. Second, the sample size required to declare statistical significance is increased as a result of the large number of hypotheses tested. Third, a majority of these studies investigate complex diseases, in which each disease variant confers a moderate risk. Thus, assuming an odds ratio of 1.5 and an allele frequency of 0.2, more than 1000 cases and controls are required for a statistical power of 80% and a significance level of *p *< 10^-6 ^[2].

Several approaches are being developed to improve the efficiency of genome-wide association studies. One attractive solution is a multi-stage design, in which only a subset of individuals are genotyped at the full set of markers in the initial phase. In subsequent stages, additional individuals are genotyped at increasingly restricted marker sets [3]. In the context of family-based association testing, methods have also been developed that use the same data for genomic screening and replication [4]. Both of these methods focus on reducing the number of hypotheses tested.

As an alternative, we note that association studies of some diseases, such as rheumatoid arthritis (RA), are being performed by more than one group of investigators, giving rise to multiple data sets [5,6]. One major challenge is that subjects from different studies, or within a study over the study period, may be genotyped by different platforms; therefore, different sets of single-nucleotide polymorphisms (SNPs) are assayed. In linkage analysis, it is relatively straightforward to combine families genotyped at different marker panels: a LOD score at an unobserved marker may be evaluated using neighboring observed markers through multipoint interval mapping techniques [7]; LOD scores at corresponding genome locations can then be added across families. In contrast, to our knowledge, a multipoint interval mapping approach has not been developed in the context of linkage disequilibrium (LD) association studies. This is because without a priori knowledge of LD patterns between markers, it is difficult to infer LD between disease and an unobserved marker based on neighboring observed markers. Furthermore, haplotypes constructed on each study cannot be easily combined. On the other hand, there is evidence that strong LD exists among tightly linked markers, and therefore we expect the test statistics at these markers to be correlated [8].

In this paper, we propose a novel approach that allows us to increase the effective sample size by combining data obtained from several studies. Specifically, we consider a situation in which both the subjects and marker panels are non-overlapping among the studies. In this situation, it is not possible to evaluate LD between markers from different panels. Our approach, which exploits the local LD structure without assuming an explicit population model, opens up the possibility of improving statistical power by incorporating existing data into future association studies. We illustrate our method by analyzing the simulated RA data. We had no knowledge of the "answers" at the time of analysis.

## Methods

We have previously described a multipoint transmission-disequilibrium test (TDT) method that is based on local smoothing [8]. Our study demonstrated that a) TDT statistics at tightly linked markers are correlated, and b) when tightly linked markers are genotyped, the smoothed TDT statistics can achieve a greater statistical power compared with the non-smoothed version. These findings suggest that TDT statistics can be combined, even though the different studies have genotyped non-overlapping set of markers.

### Combining data sets with non-overlapping markers and individuals

In this section, we outline our statistical methodology in a simple setting: two studies have genotyped non-overlapping sets of markers on independent sets of individuals in a common genomic region, and both studies have used a case-parents trio design. In each study, the TDT statistics, TDT^*A *^or TDT^*B*^, can be computed at the genotyped markers [9].

To motivate our test statistic, we first consider a marker that has been genotyped in both studies. In Study A, let *b*_1 _denote the number of informative transmissions, in which **A **alleles are transmitted but **a **alleles are not transmitted, and let *c*_1 _denote the converse (i.e., **a **alleles but not **A **alleles are transmitted). Likewise, let *b*_2 _and *c*_2 _denote the corresponding numbers of informative transmissions in Study B. With complete genotype data, we would compute the TDT by pooling the data:

$$\begin{array}{ll} T_{pool} & =\frac{{\left(b_{1}+b_{2}-c_{1}-c_{2}\right)}^{2}}{b_{1}+b_{2}+c_{1}+c_{2}}\\ & =\frac{{\left(b_{1}-c_{1}\right)}^{2}}{b_{1}+c_{1}}\frac{b_{1}+c_{1}}{b_{1}+b_{2}+c_{1}+c_{2}}+\frac{{\left(b_{2}-c_{2}\right)}^{2}}{b_{2}+c_{2}}\frac{b_{2}+c_{2}}{b_{1}+b_{2}+c_{1}+c_{2}}+\frac{2(b_{1}-c_{1})(b_{2}-c_{2})}{b_{1}+b_{2}+c_{1}+c_{2}}\\ & =\frac{n_{1}}{n_{1}+n_{2}}TDT^{A}+\frac{n_{2}}{n_{1}+n_{2}}TDT^{B}+\frac{2(b_{1}-c_{1})(b_{2}-c_{2})}{b_{1}+b_{2}+c_{1}+c_{2}}. \end{array}$$

We next show that, under the null hypothesis, the last term in Eq. (1) has an expectation of 0. Let *R*_1_* = B*_1 _+ *C*_1 _> *0 *(the capital letters denote the random variables), and *R*_2_* = B*_2 _+ *C*_2 _> 0. Under the null hypothesis, *L*(*B*_1 _| *R*_1_) ~ Binom(*R*_1_, 0.5), *L*(*B*_2 _| *R*_2_) ~ Binom(*R*_2_, 0.5), and *B*_1 _and *B*_2 _are independent. We then have:

$$\begin{array}{ll} E\left(\frac{\left(B_{1}-C_{1})(B_{2}-C_{2}\right)}{B_{1}+B_{2}+C_{1}+C_{2}}\right) & =E\left[E\left(\frac{\left(B_{1}-C_{1})(B_{2}-C_{2}\right)}{B_{1}+B_{2}+C_{1}+C_{2}}\right)|R_{1},R_{2}\right]\\ & =E\left[\frac{1}{R_{1}+R_{2}}E(2B_{1}-R_{1}|R_{1})E(2B_{2}-R_{2}|R_{2})\right]=0. \end{array}$$

Therefore, under the null hypothesis, the pooled TDT statistic is nearly a weighted average of the corresponding TDT statistics in the respective studies. The weights are proportional to the number of informative parents (*n*_*i *_= *b*_*i *_+ *c*_*i*_). Assuming that the two studies sampled comparable populations (e.g., allele frequencies are similar at all loci), we approximate these weights by the number of trios.

For a marker that is not genotyped in one study (but is genotyped in the other), we try to impute the TDT statistic using neighboring markers. We then add the observed and imputed TDT scores from the two studies. Suppose *M *markers have been genotyped by either Study A or Study B. Denote the physical locations of these markers by {*t*_1_,...,*t*_*M*_}. Let $V^{A}=[v_{1}^{A},\ldots ,v_{M}^{A}]$ be a vector indicating whether marker *m *is genotyped in Study A. Denote the TDT statistics using each of the two samples as TDT^*A *^and TDT^*B*^, respectively. Let *f*^*A*^(*t*) be the results of applying a local linear regression fitting to (*t*_*i*_, *TDT*^*A*^) data. At each marker, we compute:

$$T^{A}(t_{i})=v_{i}^{A}{\text{TDT}}^{A}(t_{i})+(1-v_{i}^{A})f^{A}(t_{i}).$$

In other words, if a marker is genotyped in Study A (*v*^*A *^= 1), we simply take the TDT statistic; if a marker *t*_*i *_is not gentoyped in Study A, we impute its expected TDT statistic using the predicted value. Similarly, we compute *T*^*B*^(*t*) using data from Study B. The combined test statistic at each marker is simply:

$${\text{TDT}}_{\text{comb}}(t_{i})=\frac{n_{1}}{n_{1}+n_{2}}T^{A}(t_{i})+\frac{n_{2}}{n_{1}+n_{2}}T^{B}(t_{i}).$$

We implement the imputation step using the loess function in R. The choice of the smoothing parameter depends on many factors such as the age of the disease mutation, the population under study, and the marker density. While an optimal window size is difficult to define, an examination of inter-marker LD guides our choice: we seek a region within which the genotyped markers are in high LD. Roughly speaking, we are faced with a trade-off between bias and variance: smoothing over a wide region tends to reduce variance of the imputed statistics at the cost of an increased bias. Therefore, an alternative to loess with pre-specified bandwidth is a smoothing spline with the degree of freedom chosen by cross-validation. To properly account for the imputation, and to correct for multiple comparison, we perform a simulation-based test: conditioning on the parents' genotype, we generate the transmitted and the non-transmitted haplotypes under the null hypothesis, re-impute the TDT statistics, and compute TDT_comb _on the simulated data. The observed max_*i*_TDT_comb_(*t*_*i*_) is compared with the null distribution of the corresponding maxima in the simulated data.

### Data set example

To illustrate our proposed method, we analyze Replicate 1 of the simulated RA data. This data set consists of 1500 nuclear families, each of which has both parents and two affected children genotyped. It is known that there is a strong effect of DR type at the HLA locus on chromosome 6. A simple TDT analysis using all 1500 families unambiguously demonstrates preferential transmission of DR-2 or DR-3 alleles to the affected individuals. However, is the DR allele the sole variant affecting the disease in the region? To address this question, we examine the transmission from parents who are homozygous 1/1 at the DR locus. If the DR locus explains the entire association in the region, conditioning on parents being 1/1, there should not be preferential transmission at any markers nearby. Among 1500 mothers, 70 have genotype 1/1. Our analyses highlight a practical difficulty: performing stratified analysis on a subset of samples further reduces the sample size; thus, stratified analyses are particularly likely to suffer from small sample size even when the main study has good power.

On chromosome 6, we restrict ourselves to the 293 SNP markers falling within 1.5 × 10^6 ^bp around the DR locus. We consider a situation in which each third of the families are genotyped on a different platform. The 293 SNPs are randomly divided into three sets, and there is no overlap in the three sets of markers or individuals. Because the risk of RA is much higher among women, we hypothesize that there may be gene × sex interaction. Furthermore, there has been ambiguous evidence regarding maternally transmitted risk elements [10]. Therefore, we looked at four types of transmission: father to son, father to daughter, mother to son, and mother to daughter. Because the phase is known for all the affected children, the four types of transmission can be examined independently. For each type of transmission, we perform a TDT analysis on each of the three subsets of families. Because the diagnosis of RA is often ambiguous, we hypothesized that the more severe cases are more likely to carry the genetic risk factor. Therefore, a severity measure, on the scale of 1 to 5, is used as a relative weight. We then combine the three sets of TDT scores to compute TDT_comb_, with a bandwidth approximately 15 markers.

## Results

**Figure 1:**
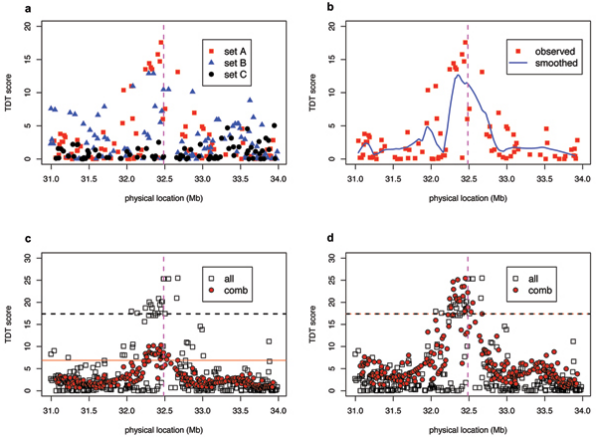
**TDT tests for mother-daughter transmissions, restricted to mothers with DR genotype 1/1**. a, TDT for three subsets separately. Dotted vertical line indicates the location of DR locus. b, TDT scores using sub-sample A (square) versus imputed scores (line). c, TDT scores assuming all markers are genotyped in each individual (TDT_all_), open square) versus TDT_comb_. Dotted line indicates 0.01 critical value for TDT_all_, and solid line represents the corresponding critical value for TDT_comb_. d, Comparison of TDT_all _and TDT_comb _after a quantile transformation.

The results of various TDT test for the mother-daughter transmission in the 3-cM region are shown in Figure 1. Figure 1a displays the TDT scores using each subset of one-third of the families. Because the transmissions from a mother to two daughters are independent, a meaningful measure of sample size is the mother-daughter pairs. In our data, the numbers of mother-daughter transmissions in the three subsets are 46, 39, and 23, respectively. For all TDT tests, we use 10,000 permutations to establish the null distribution and significance level. The *p*-values of TDT on the three subsets are 0.0011, 0.02, and 0.61, respectively. In Figure 1b, the points represent the TDT scores from subset A (families 1–500) and the solid line represents the loess prediction, ${\text{TDT}}_{\text{comb}}(t_{i})=\frac{n_{1}}{n_{1}+n_{2}}T^{A}(t_{i})+\frac{n_{2}}{n_{1}+n_{2}}T^{B}(t_{i}).$. Figure 1c compares the TDT scores when all markers are genotyped in every individual (TDT_all_, open square) versus TDT_comb _(filled points). While the maximum value achieved by TDT_comb _appears substantially lower than the corresponding value by TDT_all_, the same is true under the null hypothesis, because the imputed TDT statistics tend to be smoother than observed ones. As a result, at a specific significance level (say, 0.99), the critical value for TDT_all _is 17.38, while the corresponding critical value for TDT_comb _is 6.87. We perform a quantile transformation based on the null distribution, and Figure 1d compares TDT_all _with transformed TDT_comb_. It indicates that, upon suitable transformation, TDT_comb _can achieve similar significance level as if TDT_all_. However, the location of the peak shifts slightly: while the marker with highest TDT_all _lies to the right of the DR locus, that with the highest TDT_comb _lies to the left of DR locus. Another consequence of smoothing and imputing TDT scores is that the "peak" of TDT_comb _appears somewhat narrower than TDT_all_. In a similar fashion, we analyzed the other three types of transmission. The results, summarized in Table 1, suggest the existence of another variant that influences the disease risk. Interestingly, transmission is distorted in mother-daughter and father-daughter transmission, but not transmissions to sons. This suggests possible gene × sex interaction. Finally, while we set out to use severity as a relative weight for each individual, retrospective comparison indicates that the weight makes little difference.

**Table 1:** Results of TDT tests for sex- and parent-specific transmissions

Transmission	TDT^A^	TDT^B^	TDT^C^	TDT_all_	TDT_comb_
Mother-daughter	0.0011	0.019	0.60	<**10^-4^**	<**10^-4^**
Father-daughter	6 × 10^-4^	<**10^-4a^**	0.0023	<**10^-4^**	<**10^-4^**
Mother-son	0.056	0.241	0.77	0.159	0.0896
Father-son	0.22	0.6634	0.4440	0.68	0.62

^a^Bold indicates statistical significance at *p *= 0.05 level.

## Discussion

We have performed additional simulations to assess the gain in power of our combined test (not shown). In a wide range of disease models and parameters, the combined test achieves a greater power than the probability that at least one of the two studies achieved statistical significance, even though the latter procedure has an inflated type I error. Furthermore, the gain in power of our approach depends on marker density. In the current setting, the entire SNP set on chromosome 6 has a density comparable to a genome-wide set of 300 K, and hence each of the three subsets we analyzed has a density comparable to the 100 K SNP array. When each subset of individuals has been genotyped on a denser marker set, the imputed TDT scores tend to be more accurate. Thus, had subsets of individuals been genotyped on different platforms of 500 K SNP arrays, we would expect the imputed TDT scores to be more accurate, and therefore combining across studies will achieve even greater power. On the other hand, compared to the pooled TDT with complete marker data, the combined test incurs a loss of power. This loss of power is to be expected for two reasons. First, the smoothing process introduces uncertainties both under the null and alternative hypotheses. Second, if a locus increases the disease risk in both studies and the high risk alleles are the same allele, the last term in Eq. (1) has an expectation greater than 0; hence, E(TDT_pool_) > E(TDT_comb_).

We have developed and evaluated our approach in the context of a family-based study using the TDT design. The approach can be generalized to case-control design when participants in all samples represent a relatively homogeneous population and all studies use the exact same phenotype definition. Combining samples in the presence of population stratification requires extensions to our method, including modification on the weight and the smoothing parameters. For example, if LD among markers decays slower in one population than in the other, it maybe desirable to use a wider smoothing parameter in the former population. These issues should be examined more thoroughly in the future. On the other hand, as long as the cases and controls are matched within each study, the combined test offers greater protection against population stratification than a test on the pooled genotypes. Finally, if different genetic factors underlie the etiology of each study, combing these studies will not improve power. Therefore, an important issue to address in the future is how to decide whether different studies can be combined.

## Conclusion

Genome-wide association studies often suffer from insufficient sample size. The problem is exacerbated by the need to restrict the analysis to a subset of individuals. Our approach, which exploits the local LD structure without assuming an explicit population model, opens up the possibility of improving statistical power by incorporating existing data into future association studies.

## Competing interests

The author(s) declare that they have no competing interests.
